# Blurred transitions of female genital cutting in a Norwegian Somali community

**DOI:** 10.1371/journal.pone.0220985

**Published:** 2019-08-15

**Authors:** R. Elise B. Johansen

**Affiliations:** Norwegian Centre for Violence and Traumatic Stress Studies, Oslo, Norway; Oakland University, UNITED STATES

## Abstract

While diaspora communities have become more critical of Female Genital Cutting (FGC), there are also trends of continuity. To explore the interplay between continuity and change, I designed a study among Somali migrants in Norway. A team of six Somali research assistants collected data from 72 male and female research participants between 16 and 57 years of age through in-depth interviews and focus group discussions. The aim of the study was to gather knowledge that could improve interventions among migrant populations. The study findings indicate that the experience of FGC as a practice in transition implies that people have to maneuver between different and partly contradictory social norms. The paper first discusses the contradiction between a strong negative attitude toward FGC and very low engagement. The lack of engagement is explained by the increased privatization of FGC and insecurities due to the transition and disempowerment with regard to challenging the FGC practices of relatives based in countries of origin. Second, the paper explores the contradiction between perceptions of FGC as a disappearing practice and the recognition of trends of continuation. Trends of continuation include those related to perceptions of risk during travel to countries of origin, resistance to defibulation, support for sunna circumcision and insecurities regarding the significance of FGC for marriageability. Thus, despite an almost universally negative attitude toward FGC in the form of infibulation, ongoing changes can, to some extent, hamper further change. This suggests that to ensure further progress in the abandonment of the practice, these complex and interconnected expectations must be addressed.

## Introduction

Female Genital Cutting (FGC) is an almost universal practice among ethnic Somalis, who also traditionally practice the most extensive type commonly known as infibulation [1]. Following the civil war and unrest in Somalia in the late 1980s, Somalis fled their country in large numbers, which was followed by migration through family reunion. More than one million Somalis living outside their country of origin [2] have raised concerns about the continuation of FGC in countries of migration [3]. This paper explores perceptions of FGC among ethnic Somalis in Norway through a study exploring processes of continuity and change in an interchange between personal perceptions and sociocultural norms and conventions.

The Somalis can be described as an ethnic group traditionally inhabiting Somalia (approximately 15 million) and adjacent areas in neighboring countries, including Kenya, Djibouti and Ethiopia (approximately 15 million)[4]. Somalia itself, while internationally recognized as one country, is in reality currently divided into three autonomous states: South-Central, Somaliland and Puntland. As the last two are the most stable states, most recent studies have been carried out there.

Prevalence data on FGC among ethnic Somalis vary little across their countries of origin [5]. We thus expect the most recent prevalence studies from Somaliland and Puntland to be relatively representative of all ethnic Somalis in the horn of Africa [6, 7]. The most recent population-based data collected in 2011 found prevalence rates of 97% and 99% in the two states, respectively. The existing data also indicate very little change over time, despite a long history of interventions [5, 6, 8–12]. A common way to measure change is to compare data between older and younger cohorts. There is a slightly lower prevalence in the youngest age group (15–19) compared with the age 45–49 cohort both in Puntland and Somaliland; the differences are very small—just 0.5% lower in Puntland and 1.5% lower in Somaliland, suggesting an almost insignificant trend toward change.

Somalis traditionally practice infibulation, the most extensive type of FGC. Categorized as Type III by the World Health Organization (WHO), infibulation is described as the cutting and apposition of the labia minora and/or majora with a small opening left at the lower end for the passage of urine and menstrual blood [1]. The procedure, commonly referred to as pharaonic in Somalia, is deeply intertwined with key cultural values hinging on ideals of virginity and virtue for women and virility, sexual pleasure and paternity for men [1–3]. There is thus an intimate link between the physical extent of the procedure and its cultural meaning. The infibulated seal of the skin is understood as a culturally construed hymen, perceived as a necessary protection and proof of virginity and thus of virtue. At marriage, the seal of the skin has to be cut or torn open to enable sexual intercourse and childbirth [13].

Despite the profound cultural meaning of infibulation, however, several studies have found a growing discourse about the possibility of substituting a lesser form of cutting in Somalia, commonly referred to as “sunna circumcision” [6, 10, 12, 14]. A recent study covering 2 581 men and women across Somalia found that only 34% supported the continuation of infibulation, whereas 55% supported sunna circumcision [15]. This shift is mainly motivated by a concern about the health risks that Somalis associate with infibulation [12, 15]. In Somalia, this change is generally understood as an abandonment of FGC, as only infibulation is perceived as FGC [12, 16–18].

However, existing evidence from Somalia indicates that the understanding and definition of sunna covers a wide variety of procedures [12, 16, 17], including all four types of FGC in the WHO classification [1]. The WHO FGM typology distinguishes between: Type I—Partial or total removal of the clitoris and/or the prepuce (clitoridectomy). Type II—Partial or total removal of the clitoris and the labia minora, with or without excision of the labia majora (excision). Type III—Narrowing of the vaginal orifice with the creation of a covering seal by cutting and appositioning the labia minora and/or the labia majora, with or without excision of the clitoris (infibulation). Type IV—All other harmful procedures to the female genitalia for nonmedical purposes, for example: pricking, piercing, incising, scraping and cauterization. There are subtypes within the first three main types. The different procedures described the Somalis describe as sunna, are sometimes recognized as various subtypes, most commonly within a range from minor sunna (pricking) and small sunna (removal of tissue) to large sunna (removal of tissue followed by stitches) [12, 15, 16, 19, 20].

The limited change in the physical extent of FGC produced following the claimed change from infibulation to sunna is most likely due to the close link between the physical extent and the cultural meaning of infibulation. The traditional perception of the infibulation scar as the creation and safeguarding of virginity and virtue would suggest that performing a sunna with no or limited physical closure would fail to fulfil these values [21].

The discourse of abandonment in the form of change from infibulation to sunna has also been identified in the diaspora [9, 22–27]. In its less extensive form, such as pricking or nicking, such change has been welcomed and encouraged by some researchers and professional organizations as a form of harm reduction or a step toward abandonment [22]. However, as the understanding of sunna seems to be as blurred among Somalis in the diaspora as in their country of origin, acceptance of sunna could cover a wide variety of procedures and thus contributes to the continuation of several forms of FGC [9, 22–25, 28]. The terminology may constitute another challenge to abandonment. The term sunna is central to Islamic belief, generally used to indicate positively valued religious practices [29]. Thus, when used to describe a form of FGC, it links the practice closer to Islam, which again may be an obstacle to abandonment [15]. Furthermore, while a change in the term and type can also indicate a change in meaning, from tradition and culture to religion, there seems to be a sense of continuity in the underlying meaning that associates FGC with control of female sexuality [30].

Recent studies from Somalia have found that even though an increasing proportion of the population has stated that it wants FGC (in the form of infibulation) to end, most people are reluctant to abandon a practice that still constitutes a social convention [16]. The perceptions of FGC as a social convention have been a common theoretical framework for understanding its perpetuation despite awareness of negative health consequences and growing opposition [31]. The theory holds that, whatever the original cause of the practice, once widely practiced, it becomes locked into place as a social convention. As such, FGC becomes a sign of normality, perpetuated mainly to conform. Social conventions are upheld through social norms that are enforced through positive and negative sanctions. In FGC practicing communities, girls and women without FGC have been reported to be subject to intensive peer pressure, such as harassment, social exclusion and being considered unmarriageable [24, 31–33]. Thus, to secure a good life for one’s daughters, many families subject them to FGC, even if they might personally have a negative attitude to the practice. Thus, the continued high prevalence of FGC in Somalia indicates that there have been minimal changes with regard to FGC as a social convention.

Recent studies among Somalis in the diaspora have found that they express a stronger and broader negative attitude to FGC than Somalis in their country of origin, indicating an overall sense of FGC abandonment [23, 34–36]. While authorities in many countries of migration have expressed a concern about the risk of so-called “holiday cutting”, there are few reported FGC incidents [37–40]. This has been interpreted as an indication of a changed social convention [26, 41]. Increased knowledge of health risks, space to voice concerns and question the practice, laws against FGC and distance from the country of origin are other factors believed to contribute to this change [24, 42, 43]. Of these factors, distance from the country of origin and time of residence in the diaspora have been understood as the most influential [23, 27, 34, 42, 44].

Despite such abandonment and low support for FGC, studies have also documented a sense of continuity in the underlying cultural values among Somalis in the diaspora [45, 46]. A study including Somalis in Norway found a prevailing strong association between infibulation and its underlying cultural values relating to virginity and virtue [21]. These were the main reasons why premarital defibulation was perceived as socioculturally unacceptable, despite the fact that the procedure could ease many negative health consequences of infibulation and the negative attitude toward infibulation. The broad support for or acceptance of sunna circumcision among Somalis in the diaspora also constitute a form of continuation. Thus, an understanding of change in diaspora communities would allow for a wider complexity as to whether FGC is supported or practiced.

There is also a need for a more nuanced approach and the widening of the scope of society and social norms beyond the country of origin and the country of migration. Somali migrants in Norway relate to expectations from relatives and friends in various countries, both in the global south and global north. They may also have to consider factors relating to onward migration or return migration [47–56]. Thus, in this study, we included a wider scope of transnational networks and movements.

This review of existing knowledge on FGC discourses and practices among Somalis at home and abroad indicates complex processes of continuity and change that cut across both time and space. Thus, to build the basis for more appropriate interventions in the diaspora, I decided to conduct a qualitative study exploring these processes of continuity and change. I chose to conduct the study among Somali migrants, as they are the largest FGC practicing group in Norway, with an estimated 17 300 girls and women subjected to FGC prior to arrival [57]. I then selected a study design that had been specifically developed to explore the interrelationship between personal opinions and social norms regarding FGC in the diaspora, originally the REPLACE Approach [58, 59]. Two other advantages of this approach are the deep engagement of community-based researchers (CBRs) in most phases of the study and the systematized dynamic between the baseline study and the development, implementation and evaluation of an intervention. Furthermore, as the approach has already been implemented in several migrant communities across several countries, including Somali migrants in The Netherlands, using the same approach was expected to facilitate cross-country comparisons [60, 61].

## Materials and methods

The study followed required ethical requirements, and was approved by the Norwegian Centre for Research Data. Approval number: 5564. All research participants were given oral and written information about the project, and could choose whether to sign a written approval (none) or to give oral consent, available eon original tapes. Our choice of study site was informed by a key factor in the theory of social convention, namely, the perception of an interlinkage between physical proximity and social norms. Studies and interventions based on the theory of FGC as a social convention in countries of origin have generally used both a geographical and relational focus. The main site of social convention has been considered the local village, whereas the role of FGC for marriageability which often happen across villages, implied the inclusion of more distant locations connected through relational bonds [31]. In the diaspora, however, the location of the main reference group for a social convention is less apparent. Would it be people with a shared ethnic identity living in the same neighborhood, town or country? Or are migrants more oriented toward their home country and relatives still living there? Or are networks based on ethnicity, religion or family ties that cut across geographical borders the most significant? To facilitate an exploration of various possibilities, we selected a geographically defined study site with a suitable population size to potentially form a local community while also exploring broader networks. Furthermore, we selected a site with few, if any, previous anti-FGC interventions. This was partly because it would be more indicative of change due to migration alone and be better suited for our plans of a subsequent intervention. The selected study site was a municipality hosting about one thousand Somali adults, including a medium-size town where the majority of the Somalis in the area lived. The town hosted an active Somali mosque that was a central reference group for most local Somalis, running regular activities for women, men, youth and children. However, there was no Somali nongovernmental organization in the region.

The study tools used were based on the guides developed by REPLACE [60]. The REPLACE guide for interviews and focus groups included the following topics: FGC terminologies and types, perception of prevalence, meaning of the practice, perceived changes, knowledge of interventions and significance of FGC for ethnic identity. Adapting the tools to the Norwegian setting, we translated the guides to Norwegian and expanded on a few themes, including gender identity, marriage ideals, the acceptability of defibulation and overall perceptions of social control and belonging. The tools were then translated to Somali and pilot-tested in both their Norwegian and Somali language versions before finalization.

The study team included the author as the primary investigator and three female and three male Somali research assistants, referred to as CBRs. Criteria for their engagement included mastery of both Norwegian and Somali languages and postgraduate training. The CBRs undertook approximately 45 hours of training in qualitative research, including ethical considerations, transcription and analysis. They were involved in almost all parts of the study, including the adaption and translation of tools, recruitment and data collection, transcription and translation of data, and primary data analysis. However, they were unable to take part in the development of the current paper.

Data collection was conducted between March and September 2018. To build rapport with the local community, we started by arranging three information meetings. Research participants were recruited through these meetings and a snowball sampling of contacts made there. Some were also recruited through the personal and professional networks of the CBRs. The criteria for the inclusion of research participants were any person above 16 years of age who identified as an ethnic Somali. We aimed to achieve a good balance between males and females and between young (lower age and unmarried) and adult people (married adults). See Table 1 for more details. As shown in Table 1, we included 72 study participants, comprising 42 women and 30 men between 16 and 56 years of age.

**Table 1:** Overview of research participants and the data-collection method.

Method	Young women	Adult women	Young men	Adult men
**In-depth Interview**	7 women 16–20 years of age	9 women 37 to 56 years of age	6 men 17 to 28 years of age	5 men 33 to 50 years of age
**Focus Group Discussion**	Two groups with a total of 12 women, 16 to 22 years of age	Two groups with a total of 14 women, 35 to 57 years of age	Two groups with a total of nine men, 20 to 26 years of age	Two groups with a total of ten men, 30 to 55 years of age
**TOTAL**	42 women		30 men	

Research participants and CBRs were paired by gender, and focus groups were divided by both age and gender. Focus group discussions were led by two CBRs, one leading the discussion and the other taking notes. Interviews and focus groups were conducted in the language preferred by the research participants, resulting in most of the adult participants being interviewed in Somali and the younger generation in Norwegian, but varying with the length of stay. In the discussions, however, informants often mixed several languages—not only Somali and Norwegian but also Arabic and English. Data collection took place in a neutral locality, commonly a local Red Cross office. In addition, two interviews were conducted at the research participants’ homes, and two were conducted at their respective school/workplace for practical reasons.

Interviews lasted from 30 minutes (some of the young men) to three hours (some of the adult women), and focus groups generally lasted from 1.5 (men) to 3 hours (women). All interviews and focus groups were tape-recorded and subsequently transcribed and translated by the CBRs collecting the data.

The majority of the research participants had been born in Somalia, with the exception of one born in another country in the horn of Africa and four born in Norway. In addition, several of the research participants had lived in other countries before settling in Norway, most commonly in East Africa and the Middle East. Approximately one third of the young participants had come to Norway as young children (3 to 6 years of age), another third in early puberty, and the last third between six months and four years prior to data collection. Most of the adult women and men had arrived as adults. The time of residence in Norway ranged from six months to 30 years, with an average of approximately 10–15 years. All young research participants were students and about half of the adults were employed and the other half were housewives or unemployed.

Each transcript was read several times, analyzed individually by the primary investigator and two CBRs, and subsequently discussed by the whole team. After a preliminary analysis, we presented the results for discussion to the study community in two validation seminars, one for young women (9 participants) and one for adult men (10 participants). Insights from this validation process were then used to finetune the final analysis. The technique used for the final analysis was informed by the framework for thematic analysis [62], using manual techniques including color codes to mark recurrent themes and a summary with key information for each participant.

To ensure anonymity, we have left details about the study participants deliberately vague and assigned common Somali names as pseudonyms. To further avoid recognition, the same person may have been assigned different names in different statements; thus, the total number of names provided may exceed the number of participants. The study followed the required ethical requirements, and the Norwegian Centre for Research Data assessed that the processing of personal data complied with data protection legislation [63].

### Some methodological reflections

The REPLACE Approach emphasizes the importance of engaging CBRs, mainly for the sake of securing buy in and community ownership of the intervention phase. However, it can also be of significance for the quality of the research phase. The qualities and qualifications of the researcher are important for building the trust necessary to secure good quality data in qualitative studies. In this regard, common ethnic origin is often expected to be an asset through factors such as shared minority status, common language and cultural understanding [64]. However, several researchers have questioned whether shared ethnicity is sufficient or even always an advantage in studies on sensitive topics, as discussed by the Somali social anthropologist Kusow [64]. In studies among Somali migrants, one could expect that other factors, such as clan, education, age, social class, marital status, and time of residence in Norway, can influence the researcher/research participant relationship and thus data collection. One of the factors that seemed to affect our study was the sense that the relatively young age of the CBRs (24 to 33 years of age) facilitated our access to the youth compared to former studies conducted by the principal investigator. On the other hand, they sometimes found it difficult to obtain in-depth information from adult research participants. A major positive output of engaging ethnic Somalis in the study was that this was highly appreciated by the research participants, who communicated that they experienced this as a sign of respect and being taken seriously and involved in an issue affecting them. The young students in particular said that this inspired them to do further studies and work.

Another factor that likely affected the study was the choice of holding the first information meeting at the local Somali mosque. Though many research participants were recruited through other channels, we suspect that the proportion of religiously active members was on the high side.

### Maneuvering between contradicting social norms

Our study confirmed previous studies that found a strong and widespread negative attitude toward FGC among Somalis in the diaspora. Further discourses, however, were blurred. Study participants had varied and to some extent contradictory perceptions of social norms, which we will discuss in terms of two sets of closely interlinked contradictions. The first contradiction explored is why the strong negative attitude to FGC is associated with minimal engagement against the practice. The second contradiction discussed is between the perception of FGC as a declining practice on the one hand and strong elements of continuation on the other.

### Negative attitude and disengagement

Almost all research participants expressed a strong negative attitude toward FGC, which they considered shared within the Norwegian-Somali population. Neither did they believe that Somali men raised in Norway expect a circumcised wife. Almost all explained their negative attitude toward FGC with reference to the associated pain and health risks, of which most of the adult women and men had broad knowledge. Another common argument was a perception of FGC as a thing of the past, a disappearing tradition with no religious foundation.

Nevertheless, only few of the research participants actively promoted the abandonment of FGC. The few who had done so were two women who had intervened to discourage the cutting of younger female relatives in their country of origin and a man who had been engaged in organized anti-FGC work prior to migration. Most of the others expressed avoidance, silence and disengagement, which they linked to several interrelated factors: a new sense of FGC as a private topic, insecurities due to ongoing changes, resistance to male involvement, and a sense of disempowerment with regards to confronting FGC in the country of origin.

A new sense of privatization was striking in the comparisons that research participants highlighted between the status of FGC in Somalia and Norway. They portrayed FGC in Somalia as a social norm, in which the FGC status of girls and women was common knowledge. Girls could compare their cut genitals, and girls suspected of immoral behavior would be challenged to expose their genitals for inspection. Girls who remained uncut after a certain age were subjected to so much harassment that they would eventually beg to be circumcised. If their parents were reluctant, grandmothers were expected to take charge and organize the procedure. The uttermost shame would be a bride failing to prove her virginity through a lack of infibulation, which would most likely result in a shameful dissolving of the marriage.

In contrast, FGC in the diaspora was portrayed as a private matter only known to the immediate family. Some of the participants insisted that as a personal matter, FGC was something women could decide for themselves when reaching the appropriate age of 16 (age of sexual and medical maturity in Norway) or 18 (age of legal maturity). They further considered it inappropriate to enquire about the FGC status of others in the diaspora, including the men about to be married. Furthermore, most respondents considered it inappropriate to try to influence the decision making of others regarding FGC, including family members in other countries.

One consequence of this new sense of FGC as a private and silent issue was a renewed resistance to male involvement. Many of the research participants, both male and female, insisted that men should not talk about FGC, neither in their roles as fathers nor as religious leaders. This was generally explained as a taboo against men talking about female genitalia. One example is Omar’s opinion regarding the role of fathers. A father himself, Omar argued that

“*It would be inappropriate if the father was to talk about or interfere in discussions about the circumcision of his daughters and their genitals*. *It’s only mothers*, *grandmothers*, *aunts and other women who have anything to do with this topic*”.

The resistance to male involvement also encompassed men as religious leaders. Religious leaders were not perceived as engaged against FGC, nor were they expected to be. The reasons were similar to those discouraging the involvement of fathers: a sense of shame about men talking about women’s private parts. Anisa, a woman in her early forties, argued against male involvement this way:

“*There are almost no sheiks talking about female genitalia*, *whether he wants to or not*. *That is seen as shameful*. *He should pray and fast and stick to his own*. *He should not care about FGC*. *Neither would a chief from Norway talk about FGC directly*. *It is not his cup of tea*”.

Another argument mentioned by some was the view that FGC should stay a woman’s issue, as indicated by Suad: *“Men have nothing to do with this anyway*. *It’s not their business*. *It is the women who have the right and duty to decide for their children*”.

This resistance to male involvement conforms to traditional gender roles [33], as FGC has traditionally been women’s responsibility. Nevertheless, we had not expected this to be such a pervasive conviction among our research participants. Male involvement has been reported to increase negotiations of abandonment [5, 65, 66], which was confirmed in this study through the stories of two informants who claimed to have escaped FGC mainly due to interference from their fathers. There are also several studies that have identified significant changes in gender roles in the diaspora [30, 67–69]. Increasing male involvement, both as fathers and religious leaders, has also been argued to be key to the success of interventions [70]. Furthermore, our adult male participants demonstrated extensive insight into the health complications associated with infibulation.

How then should we understand this resistance to male involvement? One possible understanding is to interpret it as a revitalization of traditional gender roles. From a male perspective, such resistance could be seen as a way to keep or regain a sense of male authority, which some studies have found Somali men to lament that they have lost in the diaspora [69, 71]. Women’s resistance to male involvement could similarly be interpreted as a way to hold on to one of their few traditional areas of decision-making power. It could also, however, be interpreted as an aspect of the new privatization of FGC.

Another element in this apparently new form of privatization and silencing of FGC seemed to be caused by a sense of insecurity due to a sense of FGC as a practice in transition. This transition implies a divergence in FGC practices and opinions, which particularly the youth talked about as a source of insecurity and silence. As they could not know the FGC status of relatives and friends, they were concerned that raising the issue could expose themselves and/or hurt or provoke others. This insecurity also affected discourses about the type of FGC. Young women expected Somali peers with FGC to deny it to avoid feeling stigmatized, as suggested by Ubah in a focus group discussion: “*Some people still practice pharaonic*, *but they would say sunna because it would be shameful to say the truth*“. In our interviews, some of the young women with sunna circumcision initially claimed to be uncut. However, as most participants only perceived infibulation as a form of FGC, such a claim may not have been experienced as a denial. However, the new sense of privatization could protect women from feeling challenged to say anything about their genital state, as suggested by Latifa in the same focus group discussion: *“In Somalia girls without FGC will be harassed by cousins and the women in the family*. *But I don’t think you would be harassed in Norway*, *because it’s a taboo and nobody talks about it*”. Furthermore, in Norway it was having FGC that was considered as causing potential shame, as articulated by Amal, one of the young women interviewed:

“*I feel that FGC is a very sensitive topic and when I talk about it*, *I try not to speak in-depth or in detail*. *But others*, *who don’t have so much experience*, *they can end up saying wrong things that hurt people*. *Girls with FGC can feel uncomfortable and different and feel pressure that they don’t share something with others*. *They put pressure on themselves*, *because they feel that they are different and as if something is wrong with them”*.

The young women in our material expressed a deep concern that feelings of shame about being different due to FGC could cause severe mental stress, a concern that has been confirmed by other studies among young Somali women in Norway [72, 73]. This interrelationship and combination of shame and privatization can prevent young circumcised girls from sharing their experiences and seeking and receiving care and support.

Overall, both the young men and women as well as adult men shared the sense that FGC is a private and silent topic, partly due to a sense of transition. This seemed to make it difficult for women to both share personal experiences and raise the topic for general discussion. Adult women, in contrast, shared personal experiences with relative ease, which was probably partly because they expected all their peers to share the same experience of FGC.

These personal and interrelational factors contributing to the privatization of FGC, however, were further spurred by a perception of a need to protect the Somali image against negative reactions from the Norwegian host society [74]. This sense of protective denial was, for example, expressed by insisting that FGC is a long-abandoned practice in Somalia relegated only to remote rural areas and through negative comments about Somalis talking publicly about FGC in Norway. This sense of shame vis a vis the Norwegian host society, and possibly also other Muslim immigrant communities that do not practice FGC, is most likely a response to the focus on both Somali migrants and the practice of FGC in Norwegian media over the last twenty years. When I first started studying this topic in the late 1990s, this was not a concern [24]. At the time I had to abandon my initial focus on FGC as a source of shame and stigma, as it did not resonate with the concerns of research participants. While many Somalis in Norway have expressed that the public focus on FGC has encouraged their resistance to the practice [24], if the shame brought about by a negative focus contributes to increasing the privatization and silencing of the discourse, this might reverse the formerly documented increased openness to discussing the topic.

The feelings of shame and stigma were unevenly distributed among the research participants. Young women with FGC expressed a sense of shame about it and some of the young men and most of the adult men were mainly concerned with the public image of Somalis, whereas adult women first and foremost expressed a concern with the practice itself, rather than its potential stigmatizing effect.

The silence and denial of FGC discussed above further seemed to cause a sense of distrust of people’s sincerity when stating a negative view of FGC. This was particularly the case for many of the young women, who often believed that older men only pay lip service to Norwegian policy in the interest of preserving their image. One young woman, for example, claimed that some of the men talking against FGC had their own daughters cut, whereas two other young women believed that older men’s resistance to FGC would be abandoned immediately if they were to return to Somalia. Thus, the perceived desire to present a positive image of Somalia vis a vis the Norwegian host community can create doubts about their honesty. This can hamper trust and an open discussion of abandonment.

Overall, however, research participants believed that FGC had been abandoned in Norway and thus felt interventions to be superfluous. In contrast, most believed FGC to be ongoing in Somalia and insisted that this is where interventions were needed. Despite this, few tried to affect FGC decisions in Somalia. Two of the adult women had discouraged the circumcision of relatives both in the diaspora and their country of origin, whereas one of the men had been engaged in an NGO working against FGC before leaving Somalia. At the time of the study, however, no research participants took part in any anti-FGC work and had little experience or knowledge of interventions both in Norway and Somalia.

Paradoxically, while the majority considered migration as a major driver causing their resistance to FGC, they also asserted that their migrant status disqualified them from interfering in FGC decision making among those who had stayed behind. One of the adult women, Anisa, expected anti-FGC arguments to be dismissed and met with sarcasm: “*They would just say*. *Oh*, *so you are civilized now*. *Using the terms and language of the goody whites and nonbelievers*”. This feeling was even stronger among the young participants who also expected to be dismissed because of their young age. Questioning FGC in Somalia, they claimed, would be dismissed as disrespectful, breaking the important norm of showing respect for elders. A similar loss of authority to challenge cultural practices in the country of origin has also been reported from migrants in the USA [66].

These discourses of privatization, insecurities due to a sense of FGC as a practice in transition, and resistance to challenging practices and perceptions in Somalia all point to the second major contradiction between the simultaneous processes of continuation and abandonment.

### Abandonment and continuity

As indicated, many informants’ first reaction was to dismiss the necessity of talking about FGC in Norway, as it was already considered abandoned. Some also claimed the practice to be abandoned in Somalia. At the same time, however, most research participants shared stories that suggested simultaneous trends of continuation. Factors associated with continuation that will be discussed here include fear of forced FGC during travel to a country of origin, continued support for sunna, and the interrelationship between FGC and marriage.

Those who claimed FGC abandonment in Somalia generally presented this more as something that they hoped and believed was about to happen, rather than as a change that had already happened. Amira, for example, said that she believed the practice to be waning, even though the practice had been unchallenged in her community when she had left Somalia five years earlier. Khadra, who was a few years older than Amira and had lived longer in Norway, also believed the practice to be dying out in Somalia. During a recent trip to Somalia, she had met a man who was negative toward FGC and witnessed some media spots targeting the practice. Based on this Khadra concluded that “*I feel that we are about to abandon the practice (…) I feel people will get over this practice*, *if God wants*”.

At the same time, however, they perceived FGC as a pervasive social norm in Somalia. They considered elders as the main drivers upholding FGC as a social norm and as holding major decision-making power. Elders were first and foremost said to include grandmothers and other elderly female family members, as well as religious leaders and clan leaders. The perceived strong support for FGC among powerful elders implied that many research participants considered it risky to travel to their country of origin, for fear that their grandmother could have them undergo FGC. They feared that grandmothers could succeed in talking even reluctant parents into accepting the procedure or simply conducting it against their will, whether in secret or by force. One example was Anisa, a young woman who had spent half her life in Norway:

“*The grandmother has the right to cut a girl during home visits*, *and they usually go for the pharaonic type*. *There is a lot of pressure in Somalia*, *but not in Norway*. *But there can be new pressure when you travel home*, *so that those who travel can change their opinion and accept it”*.

Two of the young women had experienced this personally. During a trip to Somalia, Ebyan’s grandmother put substantial pressure on her mother to accept Ebyan to be cut. Ebyan felt that her mother was becoming persuaded, but the plans had been abandoned due to strong interference from her father.

If the reason a girl or woman stayed in Somalia was so-called “cultural rehabilitation” (Somali: dhaqan celis), this was considered to significantly increase the risk of the extended family arranging for her to undergo FGC. Dhaqan celis designates a practice whereby children and youth of both sexes are sent away to spend some time in Somalia, either in the custody of relatives or at an institution such as a school, koranic school or mental hospital [75]. Such stays are commonly motivated by a concern about youth who show signs of risky behavior in the diaspora and are meant to help by building closer rapport with their cultural identity and family. Approximately a quarter of Somali youth in the diaspora experience this [76]. Worry over their sexual morality is reportedly a common concern leading to dhaqan celis for girls and young women [76, 77]. As the meaning of FGC is closely interlinked with ideas of female sexual morality, this could increase the risk of FGC, as was suggested by Anisa: “*If the purpose of going home is to go on dhaqan celis*, *this is a confirmation that the girls need FGC*. *It confirms to the family that the procedure is needed*”.

The adult women in our sample had more varied perceptions of the risk of forced FGC by grandmothers. One example is an exchange between Leylo and Shukri during focus group discussion. Leylo insisted that there is a real risk of forced FGC, stating that *“Grandparents have a say in the future of their grandchildren*. *They have a sense of ownership of their grandchildren*. *On both sides*, *they can claim their right; I swear by Allah*, *I have heard of cases* ….”. Shukri, however, disagreed and interrupted to reject Leylo’s claim, stating that *“There is no way that could happen*. *How could anyone cut your daughter*? *It’s impossible*. *Nobody would cut the daughter without the parents’ consent*. *Not my daughter*. *I have given birth to her*, *and I decide what is good for her”*.

While Shukri and Leylo were about the same age and had lived in Norway half of their lives, in most cases, there was an association between the perception of decision-making power, age and seniority. Almost all the young women considered grandmothers to pose a significant risk of forced FGC if they were to spend a longer period of time in their custody. The young men were divided, with the ones raised in Somalia considering it to be a real risk, whereas those raised in Norway generally perceiving FGC as an abandoned practice that they did not believe affected their age mates. The Adult men in general refuted any risk. Overall, it seemed that the the older and more senior a person considered her- or himself, the less they perceived the risk of forced FGC. One woman, for example, had lost both her parents and considered herself to be the oldest generation and thus the one with the ultimate decision-making power.

A second trend in continuation was that most research participants accepted or supported sunna circumcision. While not all considered sunna as necessary, they generally described the procedure as “normal” and “harmless”, and many also believed it to be religiously condoned. As in most studies that have explored Somali perceptions of sunna, we found that the research participants had vague and varied perceptions of its physical extent. The description provided Asha, one of the adult women, was typical in the sense that it was not clear or even internally coherent:

*“Sunna is no cutting or stiches*. *You can do two stitches*, *so it’s very little*, *almost no cutting*. *Sunna is a small procedure; there is less pain and only two stitches*, *so the woman will have less pain during sex*, *periods or childbirth”*.

Most men gave even more vague descriptions, such as Ibrahim, one of the young men:

“*Sunna is to cut a small piece of the girl’s genitalia*. *It is accepted religiously*. *It is the type where you just cut a little*, *and it is less dangerous than the pharaonic*. *I have not heard of sunna posing any health risks or affecting her sexual drive”*.

Thus, there seems to be a contradiction between the widespread perception of sunna circumcision as a harmless procedure, whereas the physical extent described could be rather invasive, including both tissue removal and closure, commonly described as two or three stitches and commonly described as followed by tying the legs together. The amount of closure caused by two or three stitches and staying with tied legs until the wounds have healed was not clear. However, overall research participants considered sunna circumcision to be better than infibulation because of a less tight closure. Furthermore, all of the health risks mentioned by the participants were associated with very small vaginal introitus, including painful periods caused by obstructed flow of menstrual blood, painful sexual initiation due to tears and cuts in the infibulated scar, and birth complications due to the need for defibulation. Tissue removal in itself, or a closure that was less tight, was thus not believed to cause any complications.

A few of the women, however, feared that the change to sunna did not guarantee any change in the procedure itself, and some feared that the vague perception of sunna implied that it could be used as a cover-up for infibulation. This concern was often framed in terms of a gap between an ideal and a real sunna. Fathia, a young woman who had left Somalia in her early teens, expressed such concerns on the basis of her own experiences:

“*I have heard that sunna is a lighter type of cut*, *but it is not that different from the other type*. *I feel that nobody follows what the prophet says*. *Sunna has a larger hole than pharaonic*. *When I left Somalia eight years ago*, *pharaonic was most common*. *But you could not really see the difference*, *as girls were cut almost in the same way*, *whether it was pharaonic or sunna*. *That means it is all closed and you cannot see any major differences*. *Even the girls themselves or their parents do not know how to differentiate between the two types*. *I don’t say that circumcision is sinful*, *but the way that people do it is wrong*. *They don’t do it the way it is prescribed by religion”*.

Fathia thus thinks that there is a religiously correct way of conducting sunna, though she does not provide a description of what that would entail. Her specification of sunna entailing a larger hole, however, could indicate that she considers some extent of closure to be acceptable. There seems to be no religious script that specifies what would constitute a religiously acceptable or correct form of FGC, and religious scholars disagree as to whether some form of FGC is accepted or encouraged by Islam [29].

The change from infibulation to sunna was generally seen as a change from a cultural tradition to a religious practice. This also entailed a change in the associated meaning. Most research participants perceived infibulation as motivated by a need for sexual control, but it was considered against religion due to its health complications. In contrast, the perception of sunna as harmless implied that it was not negated by religion, and many perceived it as religiously recommended. One of the young women, Khadra, formulated the change clearly in these terms: “*Before*, *with infibulation*, *this was done to control women (sexually)*. *But nowadays with sunna*, *it is done for religious reasons*”.

The change could thus entail a fundamental cultural change, as the abandonment of infibulation would entail abandonment of the perceived need to construct a physical barrier as a protection and proof of virginity. A form of sunna that does not hinder sexual intercourse could not fulfil this role. However, virginity was still highly valued by all research participants. How was this then considered to be proven and guaranteed with a less extensive cut? None of the research participants in this study suggested that sunna circumcision, through the removal of or damage to clitoral tissue, was expected to reduce a women’s sexual drive and thus help her stay a virgin, as has been found in other studies [30]. Quite on the contrary, almost all insisted that sunna circumcision would in no way affect a women’s sexuality. Rather, increased social control over girls was mentioned as a measure that could replace the role of infibulation as a means of sexual control of unmarried girls and women.

The association between infibulation and virginity has traditionally been closely related to perceptions of women’s marriageability [25]. This was no longer perceived to be the case among men raised in the diaspora. Quite on the contrary, some research participants claimed that young men in exile preferred to marry uninfibulated women. This was believed to be motivated by their wish to avoid health complications associated with infibulation, as well as a wish for a more mutual sexual relationship. While such a shift has been applauded in intervention discourses, many young women, such as Hani, found it problematic:

“*Nowadays many men and boys say they don’t want to marry girls who have undergone FGC*. *Then think about those girls who are cut*. *The reason they were cut was that men had wanted them that way and now that has changed here in Norway*”.

Like Hani, some young girls expressed a sense of bitterness that the suffering they had undergone to be cut was no longer appreciated and could actually be a disadvantage for marriageability. However, while young women feared infibulation made them less attractive as marriage partners, they perceived premarital defibulation as completely unacceptable. Surgical defibulation can remove many of the health complications feared by men, and defibulated women could probably pass as uncut or sunna circumcised. However, there was an overall fear that defibulation would be known and interpreted as evidence of premarital intercourse and, as such, compromise a woman’s moral reputation. This puzzle needs further exploration.

Here, however, we will focus on a less explored topic regarding FGC and marriageability, namely, the implicit notion of ethnic endogamy [78]. The fear that a future husband would distrust a girl without infibulation implies that the imagined future husband is of Somali origin. To challenge this implicit notion of ethnic endogamy, we explored the acceptability of interethnic marriages. Research participants expressed a low tolerance for interethnic marriages, a view supported by available statistics. An overview of the marriage partners of women of Somali origin in Norway found that over the last years (2010–2017), 68% of their husbands were born in SomaliaAn additional 25% were born in an unspecified country other than Norway. Many of these may be ethnic Somalis, however, as half of the Somali population lives outside of Somalia. Only 3% of the women had married men of Norwegian descent (statistically defined as born in Norway to parents and grandparents born in Norway) (SSB statistics). The preference for ethnic endogamy was emphasized by all research participants, commonly referring to the perceived benefits of shared cultural and linguistic background and keeping the family close.

Some research participants, however, also described FGC as a contributing factor due to Somali men’s familiarity with FGC. Neema, for example, suggested that *“If a girl has FGC*, *it’s easier to be with a Somali who understands my struggle*”. Indirectly, FGC could also be relevant because interethnic marriages were perceived as detrimental to women’s moral reputation. If a Somali woman were to marry a non-Somali, research participants indicated that this could be interpreted as an indication of her moral failure. Her interethnic marriage would be interpreted as a result of failing to find a Somali husband, and the only reason for that could be the failure to live up to the moral standards of virginity. For example, research participants laughed at the unthinkable prospect of an infibulated woman marrying a Norwegian husband, though I have encountered several such cases in other studies [79]. More important, however, was the focus on clan endogamy and its consequences for marriage patterns.

In response to our questions on ethnic endogamy, young women often laughed, before responding rhetorically, “*How could we marry a non-Somali*, *when we can’t even marry outside our clan*”. Given the strong expectations of clan endogamy, young women did not expect to find their future partners in Norway due to our limited Somali population. The high numbers of international marriages support this impression. Thus, while the young women did not expect Norwegian-Somali men to require FGC, they were insecure about the wishes of their future husband, whom they expected to find abroad in another country with which they were unfamiliar.

## Discussion

This section discusses the study findings in light of the key elements of the theory of FGC as a social convention. This includes an expected acceleration of change, the role of social norms in upholding the convention and the definition of a community. We suggest that our study findings challenge the applicability of the theory of change in the context of the diaspora, calling for further theoretical refinements.

First, a key feature of Mackie’s theorization of FGC as a social convention relates to an expected acceleration of change. The theory assumes that individuals and families deciding to abandon FGC will declare their intention publicly and will persuade others to follow suit. Once a certain proportion of a community has done this, the community reaches a tipping point that causes the rest to follow. This happens, because when FGC ceases to be a social convention enforced through social pressure, community members will abandon the practice due to their awareness of its human costs in terms of pain and negative health consequences.

The findings in this study, however, challenge these key expectations. We found that many informants kept their views on the practice silent and rarely tried to convince others to abandon the practice. Furthermore, several research participants distrusted the sincerity of the adults speaking out against FGC, thinking that these statements were made to avoid moral and legal condemnation in Norway. Furthermore, while all the participants expressed a negative view of infibulation, the almost equally widespread support for sunna circumcision and the perception of premarital defibulation as socially unacceptable blurred the clarity of the anti-FGC statements.

These obstacles are related to different norms and values. We interpreted the silence on FGC and the lack of engagement against the practice as part of a new sense of privatization of the topic that seems to be spurred by insecurities due to ongoing changes, controversies regarding gender roles and power dynamics, and a sense of disempowerment vis a vis relatives in countries of origin.

Research participants expressed a sense of insecurity in both inter- and intraethnic relationships. Internally, there was a sense of insecurity due to diverging and unknown FGC statuses of friends and relatives, which was particularly emphasized by the youth. Externally, there was a sense of shame vis a vis the Norwegian host society, which seemed to affect both genders and age groups but was less of a concern among the adult wome Furthermore, we found increased individualization, framed partly in terms of womens individual rights to choose what to do with their own bodies and what information to share. In a sense, the social convention regarding FGC among Somali migrants in the community seems to have changed not to a convention of abandonment but to a social convention of silence and privacy.

On the other hand, participants also expressed strong social norms within the local community that directly or indirectly related to FGC. For example, expressed some of the uncut young women that they had to compensate for this by adhering more closely to social norms regarding dress code, religious engagement and general social conduct. Furthermore, whereas many informants claimed that they would accept premarital defibulation in cases of severe health problems, they considered it socially unacceptable and thus not done except in rare cases, and in these cases kept a secret. The choice of a marriage partner was also perceived as an indication of virtue, in the sense that inter-ethnic marriages were perceived as an indication of moral failure.

Our study participants often expressed a sense of struggle with the contradictory social expectations of the home and diaspora communities, fellow Muslim migrants and the Norwegian host society. For example, whereas undergoing premarital defibulation was presented as a virtual suicide if one were to travel or move back to Somalia, being infibulated was perceived as shameful vis a vis other ethnic communities. While research participants considered sunna circumcision as accepted or encouraged by Islam, they knew that many Muslim communities were unfamiliar with the practice. Moreover, whereas most informants considered migration to have played a major role in their resistance to FGC and ability to abandon the practice, they saw life in the diaspora as an undermining factor in their ability to influence FGC decisions among relatives and friends in their country of origin.

All these factors seemed to contribute to the new silencing and privatization of FGC. Thus, while the theory of social convention expects change to accelerate and even be spurred by shaming from the outside [31, 80], our findings suggest that change and perceptions of shame contributed to a silencing that seems to obstruct acceleration and diffusion of change.

A second central feature of Macie’s theory of FGC as a social convention is that the convention itself, rather than the sociocultural meaning, constitutes the main driver of change. That is, FGC is upheld mainly because everyone else does so, and if many declare a decision to abandon the practice, the rest will follow suit. Sociocultural meanings of the practice, such as paternity and sexuality, are not presented as important factors to be addressed to ensure abandonment, though he emphasizes these factors as important for the origin of FGC. Mackie suggests that FGC came into being mainly as a way to ensure paternity in a strictly hierarchical society in which rich men with several wives needed a way to ensure that their wives’ offspring were their biological children. Parents of girls would thus subject their daughters to FGC in an effort to secure a good marriage. Once the practice became settled and widespread, the society developed a perception of women’s sexuality as being in need of control, mainly as a way to legitimize the severe interference with women’s bodies that FGC entails.”*After a while*, *people in this culture begin to draw the false inference that women must be excessively wanton to require such scrupulous guarding of their honor”* [31] (p. 263). Thus, Mackie suggests that the origin of FGC was to secure paternity, and that it in hindsight became legitimized by a perception of women’s sexuality as so excessive that it became in need of control. Based on the importance that he puts on paternity and sexuality regarding the origin of FGC, it seems strange that he puts so little emphasis on these values as factors important for abandonment. Macie does suggest change to be more difficult in societies that attach strong sexual values to FGC [31](p. 279). However, whereas Mackie presents these as rare exceptions, several studies have documented FGC to be closely associated with female sexual control in most societies [46, 81–83], especially in communities practicing infibulation, in which infibulation.

Thus, one could expect anti-FGC initiatives to address the sexual concerns underlying the practice. This, however, has rarely been the case [84]. Quite on the contrary, sexuality has often been shunned as a sensitive and taboo topic and in some cases defined as irrelevant [30, 84]. I suspect that this reluctance to address sexual concerns in FGC interventions might have contributed to the resistance to premarital defibulation and the support for sunna circumcision. Sunna circumcision, while variously defined, is commonly understood to cut into the clitoris, an organ that Somalis generally perceive as the site of female sexual drive and in need of cutting to safeguard women’s sexual virtue [30]. In contrast to former studies I have conducted among Somali migrants in Norway, the participants in the current study rarely referred to sunna circumcision as a form of sexual control. This could be related to methodological factors in the study, or it could be that sexual concerns are subsumed or sublimated into religion. All our informants presented virginity and virtue of women as being of utmost religious importance, and the link between Muslim morality and sunna circumcision might be so taken-for-granted that it goes without saying.

The sole focus on FGC as a social convention per se, rather than on efforts to address the underlying sociocultural values relating to sexuality, might thus be a reason why abandonment efforts seem to have limited effect and in many cases contribute to changes in the ways in which FGC is carried out rather than to abandonment [18, 85].

A third challenge in the application of Mackie’s theory of FGC as a social convention relates to the conceptualization of the community. The theory was developed and has mainly been used to understand processes of FGC continuation and abandonment in countries of origin. Interventions by Tostan, the NGO that first tested the applicability of FGC as a social convention, emphasize the significance of public declarations of intended abandonment, both in targeted villages and in “intermarrying” villages [86]. The community upholding the social norm thus includes both the villages where people live and residents in other villages where they tend to find their marriage partners.

Among migrants, however, there is no clear delineation as to what would constitute the community framing the social convention that one relates to. In this study, research participants related to various and partially contradicting social norms from different communities. Expectations from Somalia were perceived to differ from ethnic Somalis in Norway, transnational clan-networks and the host community. Young female participants seemed to perceive the local Somali community as an important frame of reference for their daily life, such as clothing, religious engagement and social network, as well as for marriage choices and premarital defibulation. However, whereas nobody expected Norwegian raised Somalis to expect FGC of their wives, the young women did not expect to marry a Norwegian Somali. Rather, due to a perceived expectation of interclan marriages, they expected their future husband to have been raised in another country, and as such, they felt insecure as to their FGC expectations.

Thus, if one were to employ the theory of FGC as a social convention in the context of the diaspora, careful considerations would have to be made as to what constitutes significant social networks. Given the high level of transnational contact and movement, this study suggest that, at least for Somalis, family, lineage and clan networks might be as significant for change as physical proximity.

However, whereas a comparison of studies in Somalia, Norway and other countries of migrations has identified very similar discourses suggesting significant cross-country exchanges of values and views, there are also significant differences. The major difference seems to be the relationship between attitude and practice. In Norway, the negative opinions of FGC in the diaspora seem to lead to actual abandonment, even of the accepted form of sunna circumcision. In Somalia, on the other hand, infibulation seems to continue under the name of sunna, and people who claim opposition to FGC felt unable to withstand the social pressure for continuation [16]. Thus, there still seems to be a significantly larger change in FGC as a social convention in the diaspora than in the country of origin.

Finally, one of the motivations for choosing the REPLACE Approach was to facilitate cross-country comparability. Comparing our findings to those documented from the REPLACE study in the Netherlands suggests both similarities and differences [61]. A major similarity is the positive perception of replacing infibulation with sunna circumcision. A major difference is that the study in the Netherlands did not seem to find the same resistance to the engagement of men and religious leaders. Furthermore, the study participants reported more engagement as well as more awareness of anti-FGC interventions. While we have limited access to details about the study in the Netherlands, it seems that the study was conducted in communities with more experience with anti-FGC interventions and by actors engaged in these. This could be one of the reasons why they were more engaged and less reluctant to accept male involvement. If that is so, it does suggest that interventions carried out by community NGOs can significantly strengthen abandonment efforts. In Norway, most interventions are individual based, such as systematic information about the law against FGC and its health consequences by school nurses and immigration authorities [74]. Few interventions are formed as group approaches and few are minority-led [87]. This is particularly the case in the study community. In the selected municipality, health authorities, especially school nurses and midwives, have been particularly engaged in FGC issues, which mainly focus on individual encounters. In contrast, there have been almost no interventions in larger groups or initiatives led by local migrant communities. This highlights a potential challenge to anti-FGC interventions in Norway, which are highly focused on consultations with individuals, whereas the theory of FGC as a social convention indicates that group activities could be more efficient.

## Conclusion

Our study findings indicate that research participants maneuvered amid inconsistent social norms and expectations, some spurring abandonment and others hindering it. To summarize, first, the analysis explored the underlying factors that cause what seems to be a puzzling contrast between a pervasive negative attitude to FGC and the limited initiatives to encourage abandonment. Major reasons for the lack of engagement were a new sense of privatization of FGC, insecurity due to ongoing changes, controversies regarding gender roles and power dynamics, and a sense of disempowerment vis a vis relatives in countries of origin. Second, while most study participants perceived FGC as abandoned in Norway and thus saw interventions as unnecessary, they also expressed perceptions of continuation. This included acceptance of or support for sunna circumcision, insecurities as to whether future husbands would expect FGC, and a sense of risk of being forced to have FGC during travel to a country of origin.

Thus, while the study confirms former studies documenting a broad and intense negative attitude to infibulation among Somalis in the diaspora, we also identified factors that could hamper abandonment. This suggests that interventions can be beneficial in the diaspora, despite an overall negative attitude toward FGC. Furthermore, the findings suggest that if we are to develop interventions based on the theory of FGC as a social convention, there is a need for adjustments to accommodate the diaspora setting. To counteract the tendencies of silence and privatization of FGC, it would probably be useful to place an increased focus on open discussions in larger groups belonging to the same ethnic community. To counteract the tendencies of continuation, it would likely be useful to engage in broader discourses about sexuality to enable a detachment of FGC from sexual morality. This includes a need to increase anatomic and sexual knowledge and to discuss and challenge the support for sunna circumcision, as well as the perceptions underlying the resistance to premarital defibulation.

Furthermore, there is a need to strengthen diaspora communities’ ability to withstand social pressure from their country of origin, including strength to discourage FGC in the country of origin. This is a potential significant contribution to abandonment in the country of origin. More importantly, however, such engagement can be used to counteract the tendency of subjecting girls and women to FGC in their country of origin as part of preparing them for migration. Furthermore, it seems important to secure and empower young women to withstand the strong sense of vulnerability toward forced FGC during visits to their country of origin. Finally, it seems necessary to develop wider conceptualizations of what constitutes a community by including the broader perspectives of relatives and others. Influences on FGC perceptions and practices among migrants crossed ethnic, local and national boundaries, and countries of origin, countries of migration, clans, class and education. For example, the young female participants expressed a strong sense of belonging as well as social control among Somalis in the locality; however, the town and municipality hosts many other young Somali women who did not participate in our study, including young women who seemed to socialize little with other Somalis in the area. Thus, interventions would probably benefit from better coordination across countries, as well as from including discourses on cross-country influences and contrasting norms and values. Finally, it seems urgent to compensate for the almost total lack of knowledge of FGC that we identified among young men. Not only did they lack knowledge and interest, but they generally perceived FGC as a thing of the past and would thus be unprepared if they were to marry women of Somali origin, who are still likely to have undergone FGC. We did not ask about FGC status in this study, but about half of the young women participating revealed having undergone some form of FGC prior to migration, and only four of the women had been born in Norway. Thus, given the limited decline of FGC in Somali areas and through ongoing processes of migration, onward migration, family reunion and return migration, the majority of Somali women in the diaspora are likely to have undergone FGC.

## Supporting information

S1 Text Thematic interview guide translated into English. (DOCX)

S2 Text Focus group guide translated into English. (DOCX)

S3 Text Thematic interview guide in Somali. (DOCX)

S4 Text Focus group guide in Somali. (DOCX)
