# Supplementary material for: Blurred transitions of female genital cutting in a Norwegian Somali community
Source: PLoS One. 2019 Aug 15;14(8):e0220985. doi: 10.1371/journal.pone.0220985 (PMC6695242; doi:10.1371/journal.pone.0220985)
Supplement: S1 Text — (DOCX) [file pone.0220985.s001.docx]

**Information to readers:** This is a rough translation to English of the interview guide for individual interviews. During data-collection we used a Norwegian language and a Somali language version. Data-collection was carried out by Community Based Researchers (CBR). All CBR’s had assisted in adapting the questions, pilot-testing, and re-adaptation. The questions were developed as a thematic guide to the CBR’s, and meant to be handled flexibly according to the responses from research participants. Thus, sometimes some questions listed here were not asked, and others not listed were added.

**_________________________________________**

**Information to potential informant and secure informed consent:** Provide info about the project, informed consent, confidentiality, tape recording, storage and deleting. No right or wrong answers. Withdraw at any time. Use informed consent and information sheet in their preferred language.

***Can you tell me something about your background and childhood?***

*Purpose of question: Get a soft start so you get to know each other. Get a sense of their social background, how long they have lived in Norway*.

*Follow up question 1:* About background: Describe the place you grew up, urban or rural, country, family background, and numbers of siblings, education and work of parents.

*Follow up question 2:* Tell about the first time you heard about girls being circumcised? What do people think about this practice?

1. ***Which terms for female circumcision^[[1]](#footnote-1)^ do you know and what do they signify?***

*Purpose of question:* Find out what terms to use in the interview, and explore what they associate with the different terms. Find out what different types of FGC they are aware of.

*Follow up question 1*: Which terms do you know in Somali? (E.g. pharaonic, sunna, tol, small or large sunna? Other terms?). What do the different terms refer to? How are these terms used in Norway? In your country of origin?

*Follow up question 2:* If they mention pharaonic circumcision, ask them to explain its anatomical extent. Why is it called pharaonic? When and why did the Somali^[[2]](#footnote-2)^ start with this type of FGC? Reflections, thoughts.

*Follow up question 3:* If the mention sunna circumcision, ask them to provide an anatomical explanation of what they think it entails. Why is it called sunna? When did Somalis start with sunna circumcision?

*Follow up question 4:* Which Norwegian terms for female circumcision are you aware of? (kjønnslemlestelse, omskjæring). What is the signification of the different terms?

*Follow up question 5*: What type of female circumcision is common among Somalis? Have there been any changes over the last 20 years with regards to type of FGC? If yes, what changes and why?

1. ***What are the reasons for circumcision of Somali girls?***

*Purpose of question: To explore the motivations for the practice, if there are changes in the motivations, especially with regards to ideals relating to morality and honor*.

*Follow up question 1:* If they mentioned morality, ask if they thing FGC affect girls’ morality? If they do not say so, ask if they think FGC affect girls and women’s behavior or moral in any way?

*Follow up question 2: Are there any changes in the motivations for FGC in Somalia today compared to earlier, i.e. twenty years ago? (culture, religion, morality, tradition ???)*

1. ***Do some girls/young women in Norway experience social pressure^[[3]](#footnote-3)^ towards FGC?***

*Purpose of question*: To explore whether families in Norway are exposed to pressure towards circumcision their daughters, or whether girls and women without FGC experience pressure or harassment by people in Norway or other countries/country of origin. Also explore whether this varies according to type of FGC. Get insight into social norms related to FGC in Norway, including perceptions of defibulations (opening operations).

*Follow up question 1:* Do anyone experience pressure towards the circumcision of Somali girls and young women that live in Norway? If so, then Pharaonic or sunna? Is there a risk that grandparents or other relatives circumcise a Somali girl visiting her in Somalia?

*Follow up question 2:* Can uncut girls/women or their parents risk harassment or gossip in Norway? (from family in country of origin or people in Norway? If so, in which way?

*Follow up question 3:* Have you heard Somalis in Norway talking revealing that they do not want circumcision? In such cases, what are the reactions of others? (support or criticism? From whom?

*Follow up question 4:* How do Somalis in Norway perceive it if a young woman conducts a defibulation without having any marriage plans? Are there different views on this?

1. **Have health care providers talked to you about FGC?**

*Purpose of question:* Get insight into whether they have received information on FGC of Norwegian health care providers, and how they have perceived and experienced this.

*If yes- Follow up question 1:* Which terms did the health care providers use to describe circumcision of girls?

*If yes- Follow up question 2:* What did they say? How did you experience the situation? Did it include offer of genital examination and health care?

*If yes- Follow up question 3:* What type of health care can girls and women with circumcision is offered? What type of health care would you have liked to receive?

*If no- Follow up question 1:* If no healthcare providers have raised the issue of female circumcision, what do they think is the reason? Would they have wished the topic to be raised?

1. **Have you participated in any intervention against female circumcision?**

*Purpose of question:* To explore whether they have received information about circumcision in Norway or country of origin, and how they experienced this. Another purpose is to explore suggestions for later interventions.

*Follow up question 1:* Who organized the event? What type of event was it? How did you experience it? Was it a good method?

**QUICK QUESTIONS**

1. ***Which organizations working against FGC do you know?***

*Follow up question 1*: How are these organizations perceived by other Somalis?

1. ***What health risks can FGC cause?*** (Physical like cysts, pain, need for defibulation, other?)

*Purpose of question* is to explore what health consequences they are aware of, and whether they believe existing information about this, and whether they also perceive health risks with sunna circumcision.

*Follow up question 1:* Where did you get this information? How do you think about the quality of this information?

*Follow up question 2:* What do people say about health consequences of sunna circumcision? (please tell more about it)

*Follow up question 3:* What do people say about association between FGC and psychological problems? (Are there differences between pharaonic and sunna?) (night mares, fear, shame, embarrassment, sexual worries….)*.*

*Follow up question 4:* What do people say about the effect of FGC on female sexuality? (Increase or reduce women sexual desire? Increase or reduce pleasure? Is there a difference between pharaonic and sunna in this regards?)

*Follow up question 5*: Have FGC consequences for the men they have sexual relations with? (explain how. Is there a difference between pharaonic and Sunna?)

*Follow up question 6:* How do people manage complications after FGC? (Physical, psychological) What health consequences would lead them to seek health care?

1. **What do people in your vicinity say about the law against FGC?**

*Purpose of the question is* to explore what the research participants know about the law in Norway, what it entails, and how they see it.

*Follow up question 1:* Is the law forbidding all types of FGC or pharaonic only? (If they think sunna is also forbidden, how do they think about that?

*Follow up question 2:* Is FGC only forbidden after moving to Norway? (What if the parents have moved to Norway, but their daughter is still in Somalia? Is it forbidden to come to Norway already circumcised?)

*Follow up question 3:* Does the law also cover FGC during travels to country of origin?

*Follow up question 4:* What do you think about genital examination to check if a girl has undergone FGC?

If research participants mentioned cosmetic surgery or other procedures (e.g. tattoo or piercing) - *Follow up question5: Ask them to explore what they consider to be similar and different between these different procedures in genitalia*.

1. ***Do FGC have any consequences for whether Somali women in Norway get married or not?***

*Purpose of question* is to explore consequences of ethnicity, religion and FGC for selection f partner and accept of partner with another ethnic background.

*Follow up question 1:* Have women in Norway without FGC any problems of getting married? (Tell more. How do the husband and his family react if they are told that a wife/potential wife have no FGC? How do they react if they are told she has FGC? Is there a difference between men raised in Norway versus men raised in country of origin?

*Follow up question 2:* How are marriages between a Somali woman and man with another ethnic origin perceived? (Norwegian, Arab, Africa, Caribbean, Muslim-Nonmuslim..) Do they think FGC can affect choice of marriage partner?

1. **Who makes decisions regarding the circumcision of girls in Somalia and in Norway?**

*Purpose of question* is to explore men’s and women role, as well as parents versus other relatives’ role in decision making, and how this may change in diaspora).

*Follow up question 1:* What is the role of mothers with regards to daughters FGC? (Decide whether to cut or not? Type of cut? Stop cut? Organize? Pay?..)

*Follow up question 2:* What is the role of fathers with regards to daughters FGC? (Decide whether to cut or not? Type of cut? Stop cut? Organize? Pay?..)

*Follow up question 3:* What is the role of grandmothers and aunts with regards to FGC decision-making? (Do their role change for Somali families living in Norway?)

1. **Is FGC a procedure that makes a girl/woman more Somali?**

*Follow up question 1:* Is a girl/woman perceived as less Somali if she is not circumcised? (Pharaonic? Sunna?)

*Follow up question 2: Do you think FGC affect how girls/women perceive themselves as women?*

1. **For parents about children’s integration into Norwegian society:** Are you comfortable with having your children in an ordinary Norwegian school? Why/Why not? (if they mention sexuality education, gender mixed classes or other topics, explore why they are uncomfortable with that))

**FINAL SPEEDY QUESTIONS**

1. Mention as many advantages as you can about FGC
2. Mention as many disadvantages as you can about FGC
3. List any factors that facilitate continuation of FGC
4. List any factors that prevent the continuation of FGC
5. Are there any alternative cultural practices that can replace FGC/have the same advantages or functions as FGC?

1. In Norwegian language there are only two available terms for FGC, namely female circumcision and female genital mutilation. The more descriptive term, female genital cutting, does not exist in Norwegian language, but is used some places in this translation for simplicity. In the study, we used the Norwegian equivalent of «female circumcision» (kvinnelig omskjæring) in Norwegian, and common Somali terms as chosen by themselves but guided by the most common terms when initiating discussion (gudniinka for all types, pharaonic for type III and sunna as defined by study participants). [↑](#footnote-ref-1)
2. The term «Somali» is a common Norwegian way of referring to ethnic Somalis and migrants with Somali origin, as well as the population in Somalia (Norwegian: somaliere). It is also used for the language (somalisk). [↑](#footnote-ref-2)
3. During interviews, many research participants told about experiences of social pressure, while they at the same time rejected ever having experienced social pressure. When exploring this apparent contradiction with regards to our questioning, we realized that pressure was not defines as such in cases where they had been able to resist. [↑](#footnote-ref-3)
