# Supplementary material for: Blurred transitions of female genital cutting in a Norwegian Somali community
Source: PLoS One. 2019 Aug 15;14(8):e0220985. doi: 10.1371/journal.pone.0220985 (PMC6695242; doi:10.1371/journal.pone.0220985)
Supplement: S2 Text — (DOCX) [file pone.0220985.s002.docx]

**Information to readers:** This is a rough translation to English of the interview guide for individual interviews. During data-collection we used a Norwegian language and a Somali language version. Data-collection was carried out by Community Based Researchers (CBR). All CBR’s had assisted in adapting the questions, pilot-testing, and re-adaptation. The questions were developed as a thematic guide to the CBR’s, and meant to be handled flexibly according to the responses from research participants. Thus, sometimes some questions listed here were not asked, and others not listed were added.

**_________________________________________**

**FOCUS GROUP DISCUSSION THEMATIC GUIDE**

**Information to potential informant and informed consent:** Provide information about the project and confidentiality, using the information from the project information sheet and informed consent sheet. Explain the format of the focus group discussion, that some topics will be raised by the group leader for the group to discuss. Inform that we prefer you to discuss free among yourself, exchange thoughts and experiences in a respectful manner. There is no right or wrong answer, just share thoughts and opinions, reflections and ideas about the topics we will raise.

________________

1. ***Can you tell about how you think about being a Somali^[[1]](#footnote-1)^ in Norway?***

*Purpose of the question (reminder to the group-leaders): A soft start to the discussion. It’s also an important reference for our interpretation of people’s experiences of identity, if they feel at home, stigmatized or feel different.*

*Potential follow up question 1: How do you perceive the relationships and gender roles between men and women in Norway compared to in Somalia?*

*Follow up question 2: Your social interaction with other Somalis, is that related to where you live geograph8cally, or more related to family relations? Is there a lot of socialization among Somalis in this local area?*

*Follow up question 3: Which Somali traditions and values are important in Norway?*

1. **Which words are you aware of that describe circumcision^[[2]](#footnote-2)^ of women?**

*Follow up question 1: Which terms do you know in Somali? (e.g. pharaonic, sunna, tol, small or large sunna? Other terms?). What do the different terms refer to? How are these terms used in Norway? In your country of origin?*

*Follow up question 2: If they mention pharaonic circumcision, ask them to explain its anatomical extent. Why is it called pharaonic? When and why did the Somali start with this type of FGC? Reflections, thoughts.*

*Follow up question 3: If the mention sunna circumcision, ask them to provide an anatomical explanation of what they think it entails. Why is it called sunna? When did Somalis start with sunna circumcision?*

Which term/s would they prefer to use in the FGD?

*Follow up question 4*: Which Norwegian terms for female circumcision are you aware of? (kjønnslemlestelse, omskjæring). What is the signification of the different terms?

*Follow up question* 4: What type of female circumcision is common among Somalis? Have there been any changes over the last 20 years with regards to type of FGC? If yes, what changes and why?

1. ***What were the reasons for circumcision in country of origin, and historically?***

*Follow up question 1:* what were the perceived advantages or meaning of pharaonic circumcision? For whom was this an advantage? (the girl/her family/future husband/others?). How do you think about this?

*Follow up question 2:* what were/are the advantages or motivation for sunna circumcision? For who was this an advantage? (the girl/her family/future husband/others?). How do you think about this?

1. ***What do people say about the disadvantages of circumcising girls?***

*Follow up question 1:* What do people see as the disadvantages of pharaonic circumcision? For the girl/her family/her partner/the group? What are your thoughts on this?

*Follow up question l 2:* What do people see as the disadvantages of sunna circumcision? For the girl/her family/her partner/the group? What are your thoughts on this?

1. ***How are decisions on girls’ circumcision taken?***

*Follow up question 1*: What was the fathers’ tradition role with regards to FGC in Somalia? The mothers’ role? Grandparents? Other relatives on mothers or fathers side? Who decides? Who pay? Who make arrangements?

*Follow up question 2:* In Norway, who would be the most important participants or influencing decisions on FGC in Norway? People in Norway, neighbors, relatives? What happens in cases of disagreement between parents (mother and father, and other relatives, neighbors, friends, others? How are such disagreements solved?

*Follow up question 3:* If one is to send ones daughter to live with close family (grandparents, aunts/Uncles) or friends in Somalia for a month or longer, is there any risk that they would arrange for her to be circumcised?

*Follow up question 4:* If a Somali family in Norway does not want to circumcised their daughter, would the experience negative reactions or pressure from family and relatives in country of origin/other countries/Norway?

1. **How do FGC affect how girls are perceived?**

How would people in Somalia perceive a young woman without FGC?

How would Somalis in Norway perceive a young woman without FGC?

How do you think Norwegians (ethnic Norwegians) perceive Somali girls that are/they think have undergone FGC?

How do you perceive an unmarried woman who has a defibulation/open a pharaonic FGC without concrete marriage plans?

Do you know of anyone who has travelled to country of origin to reinfibulate?

1. ***What work against FGC are you aware of?***

*Follow up question: Make clear whether the discussion is about Norway or Somalia. How do they perceive such work? How do others react to it? Are they aware of the law against FGC? Does it cover both pharaonic and sunna? What types of interventions are carried out? What do they think is the effect of this work? What are the views of FGC interventions among religious leaders? In country of origin and Norway? Other types of leaders? (Clan elders, traditional leaders, others?)*

1. The term «Somali» is a common Norwegian way of referring to ethnic Somalis and migrants with Somali origin, as well as the population in Somalia (Norwegian: somaliere). [↑](#footnote-ref-1)
2. In Norwegian language there are only two available terms for FGC, namely female circumcision and female genital mutilation. The more descriptive term, female genital cutting, does not exist in Norwegian language, but is used in some places in our translation of the guidelines to English for simplicity. In the study, we used the Norwegian equivalent of «female circumcision» (kvinnelig omskjæring) in Norwegian, and common Somali terms as chosen by themselves but guided by the most common terms when initiating discussion (gudniinka for all types, pharaonic for type III and sunna as defined by study participants). [↑](#footnote-ref-2)
