# Supplementary material for: Blurred transitions of female genital cutting in a Norwegian Somali community
Source: PLoS One. 2019 Aug 15;14(8):e0220985. doi: 10.1371/journal.pone.0220985 (PMC6695242; doi:10.1371/journal.pone.0220985)
Supplement: S3 Text — (DOCX) [file pone.0220985.s003.docx]

Tani waa warbixin ku saabsan mashruuc la magac baxay (Replace). Waa mashruuc la doonayo in lagu ogaado aragtida soomalida Norway ka qabaan gudniinka. Waa muhim in qofku fahansan yahay waxa uu ogolaaday. Waajibka xog qarinta, duubista iyo xafidaadda wixii la duubo waa mid saran waraystaha. Aragtidada ayaa ah tan ugu muhimsan, ma jirto jawaab sax ah iyo mid khalad ah. Qofku wuu is beddeli karaa oo waa ka hari karaa barnaamijka marku doono.

______________

1. ***Wax ma nooga sheegi kartaa noloshaadii iyo koritantankagi hore?***

*Ujeedada su’aasha*: waa in si fudud loo billaabo waraysiga , si loo ogaado qaybaha bulshada ee ay ka soo jeedaan cidda la waraysanayo, muddada ay Norway ku noolaayeen iyo Inta ay le’eg tahay xogta ay gudniinka dumarka ka haystan?

1. Ma ii sheegi karta meeshi aad ku soo kortay ma miyi baa mase magaalo?
2. Wax ma noga sheegi karta qoyskiinu, inta qof ee kula dhalatay?
3. Wax ma noo sheegi kartaa heerka waxbarashada waalidkaa iyo shaqadii ay ka shaqayn jireen?
4. Ma ii sheegi kartaa markii ugu horreysay ee aad ogaatay in gabdhaha la gudo?

***2 Ereyadee ayaad u taqaannaa gudniinka dumarka, Maxayse ka dhiganyihiin micno ahaan?***

Ujeedadu su,aasha waa in la ogaado ereyada aad isticmaalaysaan inta uu wareysigu socdo, iyo qofka la wareysanayo waxa uu ereyada kala duwan ka fahansan yahay ama u yaqaan.

1. Ma ii sheegi kartaa magacyada gudniinka dumarka/gandhaha ee aad af-soomaaliga ku taqaano?

*(* ***Hvis ja spør B og c*** *)*

*B)* Maxay tilmaamayaan magacyadan kale duwan ee ad ii sheegtay? Sidee ayaa magacyadan looga isticmaala dalkii hooyo iyo Norway?

C) Ereygee ayaa ku haboon ee aad doonaysaa in aan wareysigan ku isticmaalno?

*D) Ma ii* sheegi karta magacyada gudniinka dumarka ee aad af-norwiijiga ku taqaano?

(Hvis hun/han vet det på norsk spør E).

*E)* Maxay tilmaamayaan magacyadan kale duwani?

*E)* Gudniin noocee ah ayaa soomaalida ku badan baad is leedahay?

F) 20-kii sano ee u danbeeyay wax is-beddel ah ma ku dhacay nooca gudniinka ah ee loo badan yahay?

C) Haddii uu is-beddel dhacay, is-beddel noocee ah ayaa dhacay – waase maxay sababtu?

1. ***Waa maxay sababaha gabdhaha soomaalida loo gudo?***

Ujeedadu su,aashu waa In la baaro sababta caaddada gudniinka loo sameeyo, gaar ahaan in sababahaasi ay yihiin kuwa la xiriira akhlaaqda iyo sharafta gabadha.

1. Gudniinka dumarku wax saamayn ah ma ku yeeshaa habdhaqanka iyo akhlaaqda gabdhaha? NEI
2. Ma aaminsan tahay in uu gudniinku dhab ahaantii saamayn ku yeesho akhlaaqda gabdhaha? JA spør B

*C? Wax miyaa iska beddelay sababaha gabdhaha maanta loo gudo Soomaaliya marka la barbardhigo 20 sano ka hor (diin, dhaqan, akhlaaq, caado-ahaan?)*

In la ogaado in qoysas Norway jooga ay la kulmaan cadaadis ah in ay gabdhahooda gudaan, iyo gabdhaha aan gudnayn in la caayo ama la cadaadiyo. Iyaga oo ay waxaas u geysanayaan dad jooga Norway ama dalal kale/dalkii hooyo. Iyo haddii ay taasi dhacdo in ay kala duwan yihiin gudniinka sunnada ah iyo midka fircooniga ah. In fikrad laga helo bulshadu sida ay Norway gudniinka u aragto, iyo weliba sida loo arko qalliinka gabadha dib loogu furayo.

*A) Ma jirtaa cid dareenta cadaadis ah in la gudo gabar Norway joogta*? Gudniin fircooni ah mise mid sunna ah?

*B) Gabar soomaali ah oo ayeydeed Soomaaliya ku booqatay, khatar ma ugu jirtaa gabadhaasi in ay ayeeyo ama dad kale gudaan*?

*C) Gabdhaha aan gudnayn ama waalidkood rabin in aan la gudin, ma dhici kartaa in la caayo ama ay sumcaddoodu xumaato*?

D) Samayn ma ku leeyihin dad jooga dalkii hooyo am dalal kale gudniinka gabar joogta Norway ?

E) Sidee ayay arrimahaasi u dhacaan?

*F) Ma maqashay dad Norway jooga oo si cad u sheegaya in aanay doonayn in gabdhaha la gudo*?

*G)* Falcelin sidee ah ayaa la kulmaan, taageero mise cambaarayn? Yaa taageera ama cambaareeya?

*H) Sidee loo arka in gabar ay samayso qalliin dib loogu furayo tolliinki gudniinka, iyada oo aanay gabadhu markaas ku jirin qorshe guur?*

1. **Shaqaalaha caafimaadku ma kala hadlen gudniinka dumarka?**

In la ogaado in ay warbixin ka heleen shaqaalaha caafimaadka ee norwiijiga, iyo sida ay warbixintaas u fahmeen.

*HADDII HAA*

*A) Ereygee ayay shaqaalaha caafimaadku isticmaaleen markii ay ka hadlayeen gudniinka dumarka*?

*B)* Mexey yirahdeen? Sidee ayaad xaaladdaas adigu u arkaysay? Baaritaan gudaha ah miyey sameeyeen?

*C) Caawin caafimaad oo noocee ah ayaa loo fidin karaa gabdhaha iyo dumarka gudan?*

*D)Caawin caafimaad oo noocee ah ayaad adigu la jeclaan lahayd in ay hesho*?

*HADDII MAYA –*

1. Haddii aysan shaqaalaha caafimaadka ee norwiijigu gudniinka kala hadlin, waa maxay sababtu ayaad is leedihiin?
2. Ma jeclaan lahaayeen in gudniinka lagaala hadlo?
3. **Adigu ma ka qaybqaadatay waxqabad gudniinka dumarka looga soo hor jeedo?**

Ujeedada wa in la ogaado in warbixin gudniinka ku saabsan lagu siiyay Norway ama dalkii hooyo, iyo sida ay warbixintaasi ahayd. Ujeeddo kale ayaa iyana ah in la soo jeediyo waxqabadyada danbe waxyaalihii lagu qaban lahaa.

*HADI HAA:*

*A) Yaa soo abaabula*? Maxay ahayd waxqabashadu? Sidee ayaad adigu u arkaysay hawshaas?

*B)* Ma aamminsan tahay in habkii hawsha loo qabtay uu ahaa mid habboon?

**SU’AALO KOOBAN:**

1. ***Ma ii sheegi karta magacyada ururrada aad taqaanno ee ka shaqeeya ka hor tagga gudniinka dumarka?***

Sidee ayaad u aragtaa ururradaan?

1. ***Ma ii sheegi karta dhibaatooyinka caafimaad ee uu gudniinka dumarku keeni karo?***

*Ujeeddada su’aashu waa in la ogaado dhibaatooyinka caafimaad ee ay yaqaannaan, in ay aamminsan yihiin warbixinta dhibaatooyinkaan laga siiyo iyo in ay fahansan yihiin in xataa gudniinka sunnada ah uu caafimaadka dhaawac u geysto*.

1. Xaggee ayaad warbixintaan ka heshay? Sidee ayaad u aragtaa tayada warbixinta aad heshay?
2. Farqi miyaa u dhexeeya gudniinka fircooniga ah iyo midka sunnada ah ?
3. Maxay dadku ka sheegaan dhibaatooyinka caafimaad ee uu keeno gudniinka sunnada ah?

*D)Maxay dadku ka sheegaan xiriirka ka dhexeeya gudniinka dumarka iyo dhibaatooyinka caafimaadka maskaxiyanka?*

*E)* Maxay dadku sameeyaan marka lala kulmo dhibaato uu gudniinka dumarku keenay?

1. Maxay dadku ka sheegaan saamaynta uu gudniinka dumarku ku yeesho galmada dumarka? (Gudniinku ma kordhiyaa mise wuu yareeyaa damaca galmada ee dumarka?
2. Saameyn ma kuu yeelankara ragga marka ay uu galmoonayan dumarka gudan?
3. Farqi miyaa u dhexeeya gudniinka fircooniga ah iyo midka sunnada ah?
4. **Dadka kugu dhow maxay ka sheegaan sharciga ka soo hor jeeda gudniinka dumarka?**

*Ujeeddada su’aashu waa in la ogaado waxa ay dadka la wareysanayo ka og yihiin sharciga Norway iyo waxa uu ku saabsan yahay, iyo sida ay iyagu sharciga u fahamsan yihiin*.

1. Ma gudniinka fircooniga ah oo keliya ayaa mamnuuc ah, misa xataa midka sunnada ah waa mamnuuc?
2. Haddii uu midka sunnada ah xataa mamnuuc yahay, sidee ayay haddaba dadku arrintaas u arkaan?
3. Gudniinku ma wuxuu mamnuuc yahay marka ay dadku Norway u soo guuraan, ka dib?
4. Ma wuxuu mamnuuc yahay gudniinku marka ay waalidku Norway joogan, laakiin gabdhihi weli joogan dalki?
5. *Mamnuuc miyaa haddii uu gudniinku dhaco mar safar/fasax lagu tagay dalkii hooyo*?

*G)* Sidee ayay dadku u arkaan arrinta ah in gabdhaha la baari karo si loo eego in ay gudan yihiin iyo in kale?

(-Still om kosmetisk kirurgi)

1. ***Maanta gudniinku saamayn ma ku leeyahay in la guursado ama aan la guursan dumarka Norway jooga?***

*Ujeeddada su’aashu waa in la baaro sida ay qawmiyadda, diinta iyo gudniinku u saameeyaan doorashada qofka lala noolaanayo iyo aqbalidda in lala noolaado qof qawmiyad kale ka soo jeeda*)

1. Dumarka aan gudnayn ma ku adag tahay in Norway lagu guursado?
2. Falcelin sidee ah ayay ninka iyo qoyskiisu ka sameeyaan haddii ay ogaadaan xaaska/gabadha uu ninku guursan doono in aanay gudnayn?
3. Falcelin sidee ah ayay ka sameeyaan haddii ay ogaadaan in ay gudan tahay?
4. Farqi miyaa u dhexeeya in uu ninku Norway ku koray iyo in uu dalkii hooyo ku soo koray?
5. Ma u malaynaysaan in uu gudniinku saamayn ku yeesho doorashada qofka la guursanayo?
6. Sidee ayay soomaalidu u arkaan guurka gabar soomaali ah iyo nin qawmiyad kale ka soo jeeda?
7. **Yaa gaara go’aanka gudniinka dumarka ee qoyska soomaaliga oo joga Soomaaliya ama Norway?**

*Ujeeddada su’aashu waa in la baaro kaalinta ragga, dumarka, waalidka iyo midda qaraabada kale. Iyo haddii ay kaalinta kuwaas qurbaha wax isaga beddeleen*.

1. Ya go’aamiya in gabdhaha la gudo iyo gudniin noocee ah?
2. Waalikda maxay qabtaan marka la eego gudniinka gabdhahooda? (hooyo iyo aabo)

*C)* Kaalin noocee ah ayay dumarka qarabada ah ku leeyihiin go’aanka ah in gabadha la gudo iyo nooca?

D) Kaalinta dadkaas ma ka duwan tahay midda soomaalida Norway ku nool?

1. **Gudniinku miyo muhiim u yahay gabadha somalinamadeeda?**
2. Gabadhu haddii aysan gudnayn ma waxaa loo arkaa in aysan soomaaliyad buuxda ahayn?

*B) Ma u malaynaysaa in uu gudniinku saameyn ku yeesho sida ay gabadhu isu aragto dumar ahaan?*

1. **Waalidka iyo dhexgalka carruurta ee bulshada norwiijiga ah:**
2. Side kula tahay in ay ilmahaagu dhigtaan iskoollada norwiijiga ee caadiga ah?
3. Gaar ahaan haddii ay soo hadal qaadaan waxbarashada galmada, fasallo ay wiilasha iyo gabdhuhu isku jiraan, ama wax kale: ka codso in ay faahfaahiyaan sababta ay raalli ug yihiin/aanay raalli uga ahayn

**SU’AALO AAD DEGDEG AH OO GABAGABO AH**

1. Ma ii sheegi karta faa’iidooyinka gudniinka dumarka u leyahay?
2. Ma ii sheegi karta dhibaatooyinka gudniinka dumarka u leyhaay?
3. Ma ii sheegi karta waxyaalaha fududeeya in ay caadada gudniinka dumarku sii socoto?
4. Ma ii sheegi karta waxyaalaha adkeeya in ay caadada gudniinka dumarku sii socoto ? Gi et eksemepl
5. Ma jiraan waxyaalo lagu badali karo gudninka dumarka?

**11) Wa maxay doorka hogamiyashaasha diinta ku leeyihin gudniinka dumarka?**

A) Wa maxay kaalinta hogamiyasha dhaqanka?

B) Farqi miyaa u dhexeyaa hogamiyasha Norwey jooga iyo kowa dalki jooga?
