# Supplementary material for: Blurred transitions of female genital cutting in a Norwegian Somali community
Source: PLoS One. 2019 Aug 15;14(8):e0220985. doi: 10.1371/journal.pone.0220985 (PMC6695242; doi:10.1371/journal.pone.0220985)
Supplement: S4 Text — (DOCX) [file pone.0220985.s004.docx]

**Hordhaca mashruuca**

Mahruucan oo lagu magacaabo  ( Replace) waa mid ku saabsan  xog uruurin la doonayo in lagu ogaado fikradaha kala duwan oo ay ka  qabaan soomalida Norway gudnninka gabdhaha. Warbixinta waajibka xog qarinta waa saaran waraystaha lana doonayo in la illaaliyo dhediinana. Hogaamiyaha kooxda ayaa su’aalo idin weydiin doona, ama soo qaadi doona mawduucyo laga doodi doono. Waxaan jecel nahay in aad dhexdiinna si xor ah u doodaan, oo aad fikradaha iyo khibradaha aad leedihiin si xushmad leh isu weydaarsataan. Ma jiraan jawaabo, fikrado ama aragtiyo sax ah iyo kuwo khalad ah. Fikradaha iyo aragtiyada kala duwan ee ka soo baxda doodiina ayaa saldhig u noqon doonta nuxurka warbixinta.

1. ***1 Sidee u aragtaan noloshaa soomalida norwey, marka laga hadlayo aqoonsiqa?***

*Ujeedada su’aasha waa:* In doodda si fudud lagu billaabo, si wax looga ogaado sida ay dadku u arkaan aqoonsigooda (identitet)  iyo In ay dareemayaan sidii dad gurigoodii jooga oo kale, oo aysan ahayn dad  ka soocan bulshada inteeda kale.

***Haddii loo baahdo su’aal midda hore la socota waydii:-***

1. Sidee ayaad u aragtaan xiriirka ragga iyo dumarka u dhexeeya  ama  doorkooda marka Norway la joogo ama marka loo eego Soomaaliya?
2. Xiriirka bulsho ee aad la leedahay soomaalida kale ma mid ku xiran meesha aad degan tihiin baa, mise waa mid qaraabanimo ku salaysan?
3. Xiriir sidee ah ayaa ka dhexeeya dadka soomaalida ah ee halkan degan ?
4. *Waa maxay dhaqanka iyo waxyaalaha la qiimeyo (verdier) ee muhiimka u ah soomaalida marka Norway la joogo*?

**2: Wax ma nooga sheegi kartaan ereyada aad u taqaaniin  ee tilmaamaya gudniinka dumarka?**

***Hadii loo baahdo su’aal midda hore la socota:***

- A_Ma ii sheegi kartaan magacyada gudniinka dumarka ee aad af-soomaaliga u taqaaniin?
- B Maxay tilmaamayaan magacyadan kale duwan, sideese ayaa magacyadan looga isticmaala dalkii hooyo ama Norway?

***Haddii ay soo hadal qaadaan gudniinka fircooniga ah***

C) weydii in ay sharaxaad ka bixiyaan sida uu yahay gudniinkan.

D) Waa maxay sababta loogu bixiyey fircooni?

E) Goorma ayay soomaalidu billaabeen gudniinka noocan ah, maxayse ahayd sababtu?

 Haddii ay soo hadal qaadaan gudniinka sunnada ah, weydii:

F)Waa maxay sababta loogu bixiyey sunna?

G) Goorma ayay soomaalidu billaabeen gudniinka sunnada ah, maxayse ahayd sababtu?

H) Ereygee ama ereyadee ayaad doonaysaa in aan isticmaalno marka aan maanta dooddeyno?

**3: Ma  sheegi kartan magacyada gudniinka dumarka ee aad af-norwiijiga ku taqaanno, maxayse tilmaamayaan magacyadani kale duwan?**

- Gudniin noocee ah ayaa soomaalida ku badan?
- 20-kii sano ee u danbeeyay wax is-beddel ah ma ku dhacay nooca gudniinka ah ee loo badan yahay?
- Haddii uu is-beddel dhacay, is-beddel noocee ah ayaa dhacay – waa maxay sabatu?

***4.***       ***Maxay ahaayeen sababihii gabdhaha loo gudi jiray dalkii hooyo/waagii hore?***

*A)*Maxay ahaayeen sababaha gudniinka fircooniga ah?

B)Yey gudniinka faa’iidu ugu jirtay? (gabadha/qoyska/ninka guursan doona  ama bulshada?)

*D)*Maxay ahaayeen sababaha gudniinka sunnada ah?

E) Yey gudniinka faa’iidu ugu jirtay? (gabadha/qoyska/ninka guursan doona /dad kale?)

***5.***       ***Dadku maxay dhibaatada gudniinka dumarka ku tilmaaman ?***

A)Gudniinku ma wuxuu dhibaa gabadha /qoyska/lammaanaheeda/bulshada?

*B)*Dadku sidebay u arkaan dhibaatada gudniinka sunnada ah?

C) Sideed arrintan u aragtaan adinka?

***6.***       ***Sidee ayaa loo gaaraa go’aanka marka gabadha la gudayo?***

*A)*Maxay ahayd doorka uu dhaqan ahaan aabahaha iyo hooyada-Soomaaliyeed ku lahaa gudniinka gabdhaha?

B) Ayeeyda, awoowe iyo qaraabada kale?

C) Yaa kharashka ku baxa dhiibi jiray, yaase soo abaabuli jiray?

***7****:***Marka Norway la joogo, ma deriska, qaraabada ama bulshada, yaa ugu muhiimsan marka la go’aaminayo gudniinka?**

1. Maxaa dhaca haddii aanay waalidku isku raacsanayn in gabadhooda la gudo? Sidee ayaa loo xalliyaa haddii aysan arrinta isku raacsanayn waalidka?

*B)* Haddii gabar muddo kooban loo diro dad qaraabada soke ah, ma laga yaabaa in ay kuwaasi gabadha gudan, iyagoon oogeysin walidkeed?

*C)*Reer soomaliyeed oo Norway ku nool haddii aysan doonayn in ay gabadhooda gudaan, cadaadis miyey kala kulmaan qoyskooda iyo qaraabada dalkii hooyo ama dallal kale jooga?

**8) Sidee ayay dadka Soomaaliya jooga u arkayaan gabadha aan gudnayn?**

A) Sidee ayay soomaalida Norway joogta u arkaan gabadha aan gudnayn?

B) Sidee ayaad u malaynaysa in qoomiyadaha kale u arkaan gabdhaha soomaalida oo gudan ama ay u malaynayaan in ay gudan yihiin?

C) Sidee ayaad u aragtaan in gabar aan la qabin, in qalliin lagu sameeyo oo laga furo tolmadii gudniinka ?

           D) Miyey dhacda in gabar norwey joogta oo horey looga furay tolliinka, in dalkii hooyo ku laabatoo, si mar kale loo soo tolo?

***9)***      ***Maxaad kala socotaa*** ***hawlaha looga soo hor jeedo gudniinka dumarka ee ka socda*Soomaaliya iyo  Norway*?***

1. *Falcelin noocee ah ayay la kulman dadka ku hawlan ka hortagga gudniinka?*
2. *Maxaa kala socotaan in ay jiraan sharciyo ka soo hor jeeda gudniinka dumarka, midka fircooniga iyo sunnaha?*
3. *Hawlo sidee ah ayaa la qabtaa si looga hor tago gudniinka?*
4. *Waxtar sidee ah ayay hawlahaasi leeyihiin bey idin la tahay?*

**10)**   **Adinku ma ka qaybqaadateen waxqabad gudniinka dumarka looga soo hor jeedo?**

Ujeedada wa in la ogaado in warbixin gudniinka ku saabsan lagu siiyay Norway ama dalkii hooyo, iyo sida ay warbixintaasi ahayd. Ujeeddo kale ayaa iyana ah in la soo jeediyo waxqabadyada danbe waxyaalihii lagu qaban lahaa.

*HADI HAA:*

1. *Yaa  soo abaabulalay? Ma waxeey aheed iskaa-wax-u qabso mase cid baa  maalgelisey?*
2. Sidee ayaad adinku u arkeeyseen hawshaas  mirro dhalkeeda?
3. Sidee ayaad u  arkeysaan heerka aqoonta dadka howshaas waday ?

*D)*Ma aamminsan tihiin in sida hawsha loo qabtay in uu ahaa mid habboon?

***11)***   ***Wa maxay doorka  hogamiyashaasha diinta ku leeyihin gudniinka dumarka?***

1. *Wa maxay kaalinta hogamiyasha dhaqanka?*
2. *Farqi miyaa u dhexeyaa hogamiyasha Norwey jooga iyo kowa dalki jooga?*
